# Hypogonadism in Adult Males with Prader-Willi Syndrome—Clinical Recommendations Based on a Dutch Cohort Study, Review of the Literature and an International Expert Panel Discussion

**DOI:** 10.3390/jcm10194361

**Published:** 2021-09-24

**Authors:** Karlijn Pellikaan, Yassine Ben Brahim, Anna G. W. Rosenberg, Kirsten Davidse, Christine Poitou, Muriel Coupaye, Anthony P. Goldstone, Charlotte Høybye, Tania P. Markovic, Graziano Grugni, Antonino Crinò, Assumpta Caixàs, Talia Eldar-Geva, Harry J. Hirsch, Varda Gross-Tsur, Merlin G. Butler, Jennifer L. Miller, Sjoerd A. A. van den Berg, Aart J. van der Lely, Laura C. G. de Graaff

**Affiliations:** 1 Department of Internal Medicine, Division of Endocrinology, Erasmus University Medical Center Rotterdam, 3015 GD Rotterdam, The Netherlands; k.pellikaan@erasmusmc.nl (K.P.); y.benbrahim@erasmusmc.nl (Y.B.B.); a.rosenberg@erasmusmc.nl (A.G.W.R.); k.davidse@erasmusmc.nl (K.D.); s.a.a.vandenberg@erasmusmc.nl (S.A.A.v.d.B.); a.vanderlelij@erasmusmc.nl (A.J.v.d.L.); 2 Center for Adults with Rare Genetic Syndromes, Department of Internal Medicine, Division of Endocrinology, Erasmus University Medical Center Rotterdam, 3015 GD Rotterdam, The Netherlands; 3 Dutch Center of Reference for Prader-Willi Syndrome, 3015 GD Rotterdam, The Netherlands; 4 Academic Center for Growth Disorders, Erasmus University Medical Center Rotterdam, 3015 GD Rotterdam, The Netherlands; 5 Assistance Publique-Hôpitaux de Paris, Rare Diseases Center of Reference ‘Prader-Willi Syndrome and Obesity with Eating Disorders’ (PRADORT), Nutrition Department, Pitié-Salpêtrière Hospital, F-75013 Paris, France; christine.poitou-bernert@aphp.fr (C.P.); muriel.coupaye@aphp.fr (M.C.); 6 International Network for Research, Management & Education on Adults with PWS (INfoRMEd-PWS); tony.goldstone@imperial.ac.uk (A.P.G.); charlotte.hoybye@sll.se (C.H.); tania.markovic@sydney.edu.au (T.P.M.); g.grugni@auxologico.it (G.G.); a.crino@tiscali.it (A.C.); acaixas@tauli.cat (A.C.); 7 ENDO-ERN (European Reference Network); 8 PsychoNeuroEndocrinology Research Group, Centre for Neuropsychopharmacology, Division of Psychiatry, and Computational, Cognitive and Clinical Neuroimaging Laboratory, Department of Brain Sciences, Faculty of Medicine, Hammersmith Hospital, London W12 0NN, UK; 9 Department of Endocrinology, Imperial College Healthcare NHS Trust, London W12 0HS, UK; 10 Department of Molecular Medicine and Surgery, Karolinska Institutet, 171 76 Stockholm, Sweden; 11 Department of Endocrinology, Karolinska University Hospital, 171 76 Stockholm, Sweden; 12 Metabolism & Obesity Services, Royal Prince Alfred Hospital, Camperdown, NSW 2050, Australia; 13 Charles Perkins Centre, Faculty of Medicine and Health, University of Sydney, Sydney, NSW 2006, Australia; 14 Division of Auxology, Istituto Auxologico Italiano, IRCCS, 28824 Piancavallo, Italy; 15 Reference Center for Prader-Willi Syndrome, Bambino Gesù Hospital, Research Institute, 00050 Palidoro (Rome), Italy; 16 Endocrinology and Nutrition Department, Parc Taulí Hospital Universitari, Institut d’Investigació I Innovació Parc Taulí I3PT, Department of Medicine, Universitat Autònoma de Barcelona, 08208 Sabadell, Spain; 17 The Israel Multidisciplinary Prader-Willi Syndrome Clinic, Jerusalem 9103102, Israel; gevat@szmc.org.il (T.E.-G.); hirschmd@gmail.com (H.J.H.); varda.gross@gmail.com (V.G.-T.); 18 Reproductive Endocrinology and Genetics Unit, Department of Obstetrics and Gynecology, Shaare-Zedek Medical Center, Jerusalem 9103102, Israel; 19 Hebrew University School of Medicine, Jerusalem 9112102, Israel; 20 Department of Pediatrics, Shaare Zedek Medical Center, Jerusalem 9103102, Israel; 21 Neuropediatrics Unit, Department of Pediatrics, Shaare Zedek Medical Center, Jerusalem 9103102, Israel; 22 Departments of Psychiatry, Behavioral Sciences and Pediatrics, University of Kansas Medical Center, Kansas City, KS 66160, USA; mbutler4@kumc.edu; 23 Department of Pediatrics, College of Medicine, University of Florida, Gainesville, FL 32610, USA; millejl@peds.ufl.edu; 24 Erasmus Medical Center, Department of Clinical Chemistry, University Medical Center Rotterdam, 3015 GD Rotterdam, The Netherlands

**Keywords:** Prader-Willi syndrome, hypogonadism, pituitary gland, testosterone, obesity, puberty

## Abstract

Prader-Willi syndrome (PWS) is a complex genetic syndrome characterized by hyperphagia, intellectual disability, hypotonia and hypothalamic dysfunction. Adults with PWS often have hormone deficiencies, hypogonadism being the most common. Untreated male hypogonadism can aggravate PWS-related health issues including muscle weakness, obesity, osteoporosis, and fatigue. Therefore, timely diagnosis and treatment of male hypogonadism is important. In this article, we share our experience with hypogonadism and its treatment in adult males with PWS and present a review of the literature. In order to report the prevalence and type of hypogonadism, treatment regimen and behavioral issues, we retrospectively collected data on medical interviews, physical examinations, biochemical measurements and testosterone replacement therapy (TRT) in 57 Dutch men with PWS. Fifty-six (98%) of the patients had either primary, central or combined hypogonadism. Untreated hypogonadism was associated with higher body mass index and lower hemoglobin concentrations. TRT was complicated by behavioral challenges in one third of the patients. Undertreatment was common and normal serum testosterone levels were achieved in only 30% of the patients. Based on the Dutch cohort data, review of the literature and an international expert panel discussion, we provide a practical algorithm for TRT in adult males with PWS in order to prevent undertreatment and related adverse health outcomes.

## 1. Introduction

Prader-Willi syndrome (PWS) is a rare genetic syndrome caused by the absence of expression of a cluster of paternally expressed genes on chromosome 15q11.2-q13, also called the ‘PWS region’. PWS can be caused by paternal deletion of (part of) the PWS region (60–75%), maternal uniparental disomy 15 (mUPD, 20–35%), imprinting center defect (ICD, 1–4%) or paternal chromosomal translocation (0.1%) [1,2]. Due to hypothalamic dysfunction, patients with PWS often have hormone deficiencies, hyperphagia, sleep disorders, abnormal temperature regulation and high pain threshold. PWS also has a characteristic neurobehavioral phenotype, including mild to moderate intellectual disability, autism-like features, obsessive compulsions, skin picking, and temper tantrums [3,4,5,6,7].

The most common hormone deficiency in PWS is hypogonadism. The reported prevalence of hypogonadism in adult males with PWS ranges from 57 to 100% [8,9,10,11,12,13,14,15,16,17,18,19,20]. Although hypogonadism in PWS can be the result of hypothalamic dysfunction, recent studies show that hypogonadism in PWS can also be the result of primary gonadal failure [16,17,21], or a combination of hypothalamic and gonadal dysfunction [16,22,23].

Hypogonadism can affect males with PWS at all ages. At birth and during infancy, boys with PWS can display cryptorchidism, scrotal hypoplasia and short penile length [24]. Later in life, small penile length in combination with large suprapubic fat may lead to voiding difficulties in young, obese adults with PWS [24]. Puberty is usually incomplete and delayed, although precocious adrenarche and, rarely, precocious puberty can also occur [25,26,27]. Primary testicular dysfunction is a major contributor to abnormal pubertal development in males with PWS [23]. In adulthood, individuals with PWS often have low levels of sex steroids [8,15,18,19,28,29,30]. Males with PWS are believed to be infertile and no cases of paternity have been reported in the literature [21,24].

Male hypogonadism is associated with fatigue, depression, decreased muscle strength and mass, increased fat mass, decreased sexual quality of life, and an increased risk of osteoporosis [31,32,33] and cardiovascular disease [32,34]. As many of these factors are already prevalent in PWS [7], it is important to detect hypogonadism and start testosterone replacement therapy (TRT) at an early stage. However, TRT is a delicate matter as it may be complicated by challenging behavior [26,35].

In the current article, we share our experience with hypogonadism and its treatment in a Dutch cohort of adult males with PWS. We report the prevalence and type of hypogonadism, treatment regimen and behavioral issues encountered in adult males with PWS. Based on our findings, a thorough review of the literature and the clinical expertise of an international expert panel discussion, we provide a practical algorithm for the treatment of hypogonadism in adult males with PWS.

## 2. Materials and Methods

Ethical review and approval were waived for this study by the Medical Ethics Committee of the Erasmus University Medical Center.

In this retrospective study, we included adult males who visited the multidisciplinary outpatient clinic of our PWS reference center in the Erasmus University Medical Center, Rotterdam, the Netherlands, between January 2015 and December 2020 and underwent our routine systematic health screening. As described previously (see [36]), this screening consists of a structured interview, a complete physical examination, a medical questionnaire, a review of the medical file, biochemical measurements and, if indicated and feasible, additional tests.

As part of regular patient care, primary caregivers were asked to fill out a medical questionnaire. In this questionnaire, subjective complaints (daytime sleepiness, fatigue, sexual complaints and temper tantrums) were scored on a 5-point Likert scale (1 = rarely or never, 2 = not often and/or not severe, 3 = quite often and/or quite severe, 4 = often and/or severe, 5 = very often and/or very severe). A score of 3 or higher was considered clinically relevant.

During the visit, blood samples were taken for general medical screening, including evaluation of gonadal function (luteinizing hormone (LH), follicle-stimulating hormone (FSH), total testosterone and sex hormone binding globulin (SHBG)) and the hematopoietic system (hemoglobin and hematocrit).

Before 1 February 2018, testosterone concentrations were measured using the PerkinElmer CHS™ MSMS Steroids Kit and an ultra-performance liquid chromatography–tandem mass spectrometer (UPLC-MS/MS) (reference range 10.0–30.0 nmol/L). After that date, testosterone concentrations were measured using an in-house assay and a UPLC-MS/MS (reference range 10.0–30.0 nmol/L). Before 1 February 2019, LH and FSH concentrations were measured using the Siemens Immulite 2000XPi (reference range 1.5–8.0 IU/L for LH and 2.0–7.0 IU/L for FSH). After that date, LH and FSH concentrations were measured using the Fujirebio Lumipulse G1200 (reference range 1.0–5.5 IU/L for LH and 0.8–5.1 IU/L for FSH). Before 15 June 2020 SHBG, concentrations were measured using the Siemens Immulite 2000XPi (reference range 10–70 nmol/L). After that date, SHBG concentrations were measured using the IDS-ISYS (reference range 10–70 nmol/L). Hemoglobin and hematocrit were measured using the Sysmex XN1000 analyzer (reference ranges 8.6–10.5 mmol/L and 0.4–0.5 L/L, respectively). LH and FSH measurements changed methods during the study with a different calibration, testosterone and SHBG measurements also changed methods, but they were calibrated similarly, as checked by external quality assessment schemes.

The visits to our outpatient clinic are always in the afternoon. In one visit, the patients are seen by the multidisciplinary team, after which blood is collected for general health screening. Although testosterone is preferably measured in the morning [37], in our clinic this was not feasible. Hypogonadism was defined as an afternoon total testosterone value below 10.0 nmol/L (2.88 ng/mL) with normal SHBG and sparse facial hair. Only if hypogonadism was not clearly present from clinical features (prepubertal status, underdeveloped genitals and/or absent virilization), a separate morning testosterone analysis was done to confirm hypogonadism. Pubic hair Tanner stage is often relatively advanced in PWS men due to normal or increased production of adrenal androgens, however, this does not represent testicular development or gonadal hormone secretion, and therefore, pubic hair was not considered in the diagnosis of hypogonadism [27]. Due to hyperphagia, it was not feasible to obtain fasting testosterone measurements. If patients already used TRT before the first visit to our outpatient clinic, this was also considered as indicating presence of hypogonadism. Only LH, FSH and testosterone values from before the start of TRT were included.

When TRT was initiated at our outpatient clinic, a daily dose of 10 mg transdermal testosterone gel was administered, which was increased by 10 mg every 4 weeks until serum testosterone concentrations within the normal range were reached. When adverse effects occurred, the TRT dose was not further increased or was decreased, depending on the severity of the adverse effects. After TRT was started, SHBG measurements were not routinely repeated.

We defined short-acting injections as injections that have to be administered every 1–6 weeks, and long-acting injections as injections that have to be administered every 12 weeks.

Hypothalamic dysfunction of the LH/testosterone axis was defined as a low or normal LH concentration with a low testosterone concentration, while testicular dysfunction was defined as a high LH concentration with a low testosterone concentration. Hypothalamic dysfunction of the FSH/inhibin B axis was defined as a low or normal FSH concentration, while testicular dysfunction was defined as a high FSH concentration. Inhibin B was not measured, but based on previous research, we would expect that inhibin B levels would be low in most males [16,18].

Patients that were treated by the pediatric endocrinologist at our reference center during childhood, received transitional care during transition to the multidisciplinary outpatient clinic for adults with PWS. Transitional care included a shared visit with both the pediatric and the adult endocrinologist, followed by alternating visits at the pediatric and adult department until the final transfer to adult endocrinology.

### 2.1. Literature Search

In collaboration with the Erasmus MC Medical Library, we performed a literature search on 24 September 2020 and last updated the search on 3 June 2021. We searched the following databases: Embase, Medline (Ovid), Web of Science Core Collection and Cochrane Central Register of Controlled Trials. We reviewed the literature for articles reporting the prevalence of hypogonadism and laboratory measurements (e.g., testosterone, LH, FSH, SHBG, inhibin B) in males with PWS. Search terms included ‘Prader-Willi Syndrome’, ‘gonadal disease’, ‘hypogonadism’, ‘puberty’, and relevant laboratory measurements. For the full search strategy, see Table S1. We excluded conference abstracts, non-original research articles, articles that were not available in English, and articles that included less than ten adults (males and females) with PWS. When articles reported on adults and children and the prevalence of hypogonadism or laboratory values were not available for adults only, we contacted the authors to retrieve information for the adults separately. When articles reported on overlapping populations, the article with the most patients or, when the number of patients was similar, the most recent article was included. Although this search strategy resulted in articles on hypogonadism in both males and females with PWS, only the articles that provide information on hypogonadism in males are reported here.

### 2.2. Expert Opinion

An international panel of PWS experts was asked to fill out a survey on their experience with the treatment of hypogonadism in adult males with PWS. Clinical recommendations have been made based on this survey, the results of the cohort study and the literature review. None of the experts had a financial interest in any of the modalities of TRT.

### 2.3. Data Analysis

Statistical analysis was performed using R version 3.6.3 (https://cran.r-project.org/, accessed on 16 September 2021). Descriptive statistics for continuous variables are reported as median (interquartile range (IQR)). For dichotomous variables we display the number and the percentage of people, *n* (%). For the comparison of untreated male hypogonadism compared to no hypogonadism or treated hypogonadism, we used the Wilcoxon rank sum test for continuous variables and a Chi-squared test for dichotomous variables. To correct for age, we used linear and logistic regression models, respectively, with a likelihood ratio test. To compare testosterone or SHBG concentrations between genotypes and between patients who used and did not use growth hormone (GH) treatment, we used a Wilcoxon rank sum test. To correct for age, we used a linear regression model with a likelihood ratio test. To investigate the relationship between testosterone or SHBG concentrations, and body mass index (BMI) and age, the Kendall rank correlation test was used. To explore the relationship between testosterone or SHBG concentrations, and BMI corrected to age, a linear regression model and a likelihood ratio test were used. For all analysis involving FSH and LH measurements, a linear regression model with a likelihood ratio test was used and a variable indicating whether the measurement was performed before or after 01-02-2019 (when the method was changed with a different calibration) was included in the model. As this was an exploratory analysis, no correction for multiple testing was performed. *p*-values < 0.05 were considered statistically significant.

## 3. Results

### 3.1. Baseline Characteristics

Baseline characteristics are shown in Table 1. We included 57 adult males with a median age of 29 years (IQR 20–40) (range 18–72 years). Only one patient was more than 60 years old and 21 patients were younger than 25 years old. One patient was excluded from the analysis. We did not screen for hypogonadism in this individual as TRT was unfeasible due to serious behavioral challenges that were already present before the first visit to our outpatient clinic.

### 3.2. Hypogonadism

**Figure 1:**
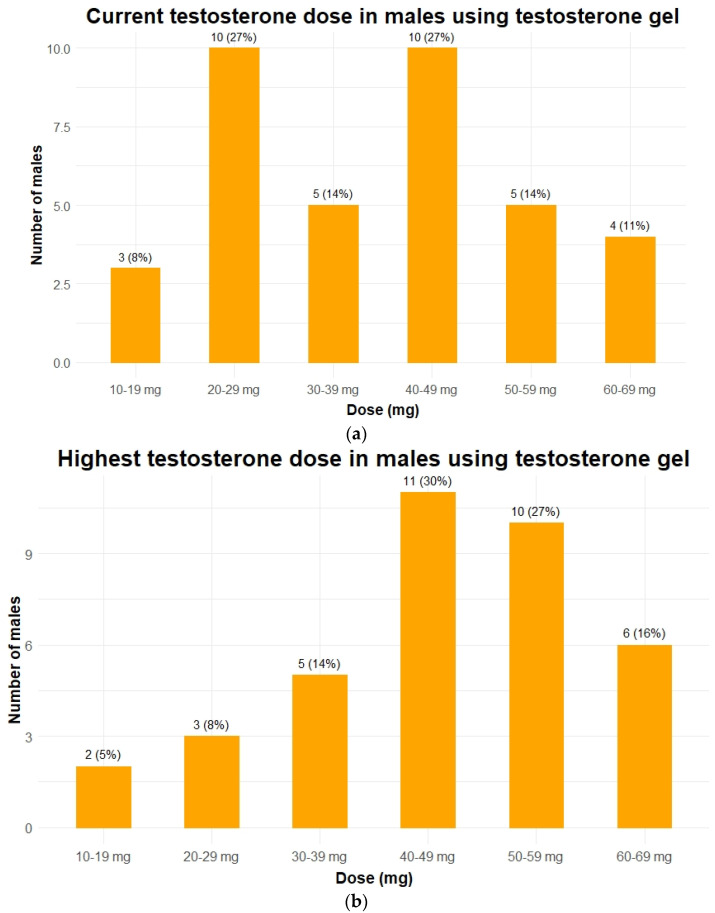
Testosterone dose in 37 males with PWS using testosterone gel. Data is given as *n* (%). (**a**) The testosterone doses each patient received during the last visit to the outpatient clinic. When patients died or were transferred to another hospital, the last known dose was given; (**b**) the highest dose of testosterone gel ever received while visiting our outpatient clinic for each patient. To make both graphs comparable only patients who currently still use testosterone gel are depicted in panel b (in 5 patients testosterone replacement therapy was discontinued completely).

Hypogonadism was present in 56 of 57 males (98%). In 28 males (49%), hypogonadism had been previously diagnosed and 24 males were already receiving TRT. Our screening revealed hypogonadism in another 28 patients (49%). Most frequent modes of testosterone administration at the first visit to our center were transdermal gel (*n* = 12, 50%) and intramuscular injections (*n* = 10, 42%). Nine males used short-acting intramuscular injections (Sustanon^®^) and one used long-acting intramuscular injections (testosterone undecanoate, Nebido^®^). For practical and/or behavioral reasons (see also discussion section), 8 patients switched from oral TRT (*n* = 2) or intramuscular injections (*n* = 6) to transdermal testosterone gel after their first visit (Table 2). The current and highest dose of transdermal testosterone gel of each patient is shown in Figure 1a,b.

**Figure 2:**
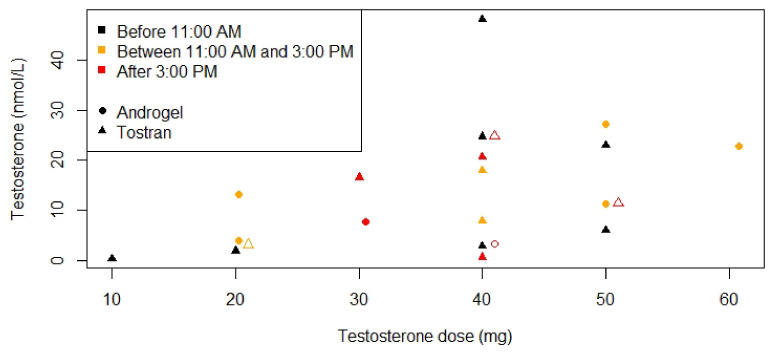
Serum testosterone concentrations according to testosterone dose for patients using testosterone gel. Laboratory measurements were only available for 22 males. Testosterone gel was administered by the patient in the morning. Testosterone measurements before 11:00 A.M. are depicted in black, between 11:00 A.M. and 3:00 P.M. in orange and after 3:00 P.M. in red. Only two brands were used; Androgel^®^ is depicted with circles and Tostran^®^ with triangles. When two points overlapped, one of the points was moved 1 mg to the right and this point is depicted with an open circle or triangle instead of closed.

**Figure 3:**
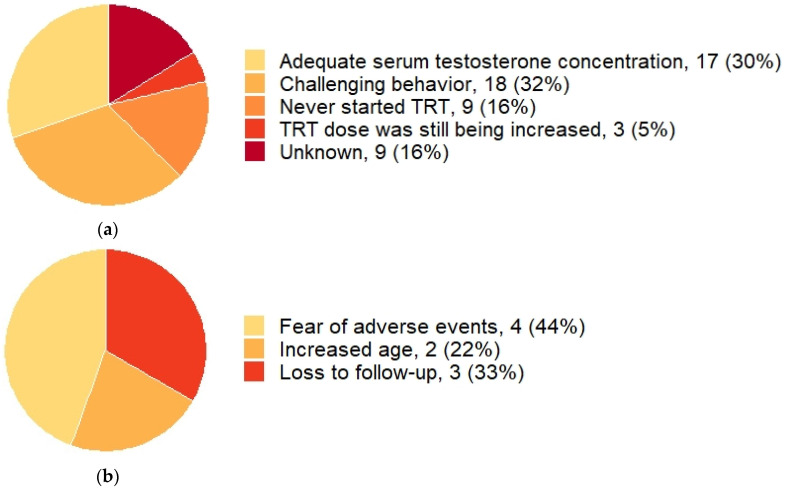
Reasons for not increasing testosterone doses and reasons for not initiating testosterone replacement therapy. Abbreviations: testosterone replacement therapy (TRT). Data is given as *n* (%). (**a**) The reasons for not further increasing testosterone doses in adult males with PWS and hypogonadism (*n* = 56). (**b**) The reasons for never initiating TRT in adult males with PWS and hypogonadism (*n* = 9).

Figure 2 shows serum testosterone concentrations according to the current testosterone dose in 22 males. This figure shows that while higher testosterone doses lead to higher serum testosterone concentrations, low testosterone concentrations can also be seen in patients using higher doses of testosterone gel. Serum testosterone levels in the normal range were reached in 17 (30%) patients. Of the 70% not reaching normal range testosterone levels, 9 patients never started TRT at all (Figure 3a), due to fear of adverse events (*n* = 4), increased age (*n* = 2), or loss to follow-up (*n* = 3) (Figure 3b). In 27 males, TRT dose could not be increased, either due to challenging behavior (*n* = 18) or for unknown reasons (*n* = 9). Three (5%) patients had inadequate testosterone doses because they were still gradually increasing testosterone dose at the time of publication of this manuscript.

In 18 patients, testosterone dose had to be decreased due to challenging behavior, of whom the majority (83%) had serum testosterone concentrations below the reference range. Seventeen of them used transdermal gel (10 mg daily, *n* = 3; 20 mg daily, *n* = 2; 30 mg daily, *n* = 2; 40 mg daily, *n* = 2; 50 mg daily, *n* = 5; 60 mg daily, *n* = 2; and 69 mg daily, *n* = 1) and one used short-acting testosterone injections (200 mg every 4 weeks). In 11 (61%) behavior improved after testosterone dose reduction.

In 5 patients TRT was stopped completely, because even 10 mg transdermal testosterone gel was followed by unacceptable behavioral challenges (*n* = 4) or depressive symptoms (*n* = 1).

Problems with compliance were fairly common, with non-compliance confirmed in 6 patients (11%) and suspected in 5 (9%).

Eight males, of whom two used short-acting testosterone injections and six with untreated hypogonadism before screening, had enlarged breasts, either due to gynaecomastia or lipomastia. In the two patients using TRT it was unknown whether breast enlargement was related to TRT. Six patients with enlarged breasts had obesity and two were overweight. In the other 49 males, the medical records did not mention gynaecomastia or lipomastia. Estradiol levels were not available.

### 3.3. Effect of Untreated Hypogonadism

We compared males with and without untreated hypogonadism at the first visit to our outpatient clinic. After correction for age, we found a significant difference in BMI (median (IQR): 29 kg/m^2^ (27–38) in males with untreated hypogonadism and 26 kg/m^2^ [23,24,25,26,27,28,29,30,31,32,33,34,35,36,37] in males with treated or no hypogonadism, *p = 0.001*). Three patients (12%) with treated or no hypogonadism had obesity, compared to 14 (44%) patients with untreated hypogonadism. Hemoglobin was significantly lower in males with untreated hypogonadism (median 8.2 nmol/L (IQR 8.0–9.0)) than in males with treated or no hypogonadism (median 9.3 nmol/L (IQR 8.6–9.7), *p = 0.03*). Although not significant, anemia was less prevalent in patients with treated or no hypogonadism (*n* = 4, 17%), compared to patients with untreated hypogonadism (*n* = 11, 34%). After correction for age, subjective complaints did not differ between the males with untreated hypogonadism and the males with treated or no hypogonadism (Table 3).

**Figure 4:**
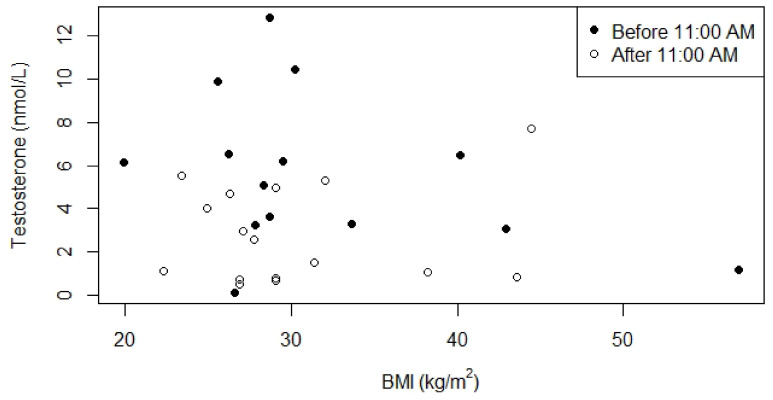
Relationship between serum testosterone concentrations and BMI for males who were not receiving testosterone replacement therapy. *p*-value for the relationship between BMI and serum testosterone concentration measured before 11:00 A.M. was 0.4 (0.1 after correction for age), Kendall’s Tau was -0.19. *p*-value for the relationship between BMI and serum testosterone concentration measured after 11:00 A.M. was 0.9 (1.0 after correction for age), Kendall’s Tau was 0.03.

**Figure 5:**
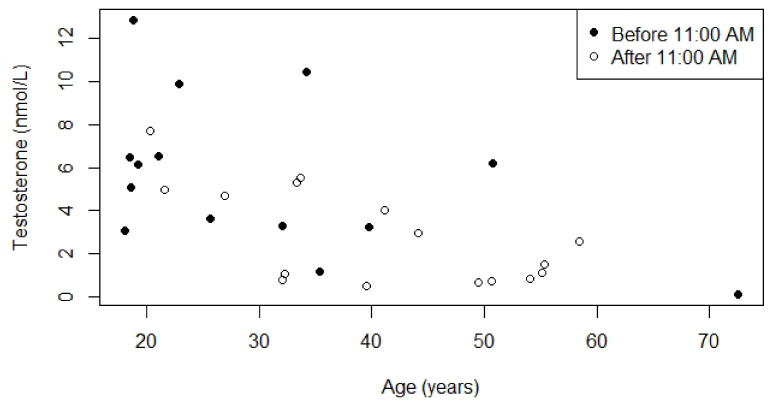
Relationship between serum testosterone concentrations and age for males who were not receiving testosterone replacement therapy. *p*-value for the relationship between age and serum testosterone concentration measured before 11:00 A.M. was 0.3, Kendall’s Tau was −0.23. *p*-value for the relationship between age and serum testosterone concentration measured after 11:00 A.M. was 0.2, Kendall’s Tau was −0.27.

We investigated the relationship between testosterone, BMI and age (Figure 4 and Figure 5) because in the normal population these parameters affect serum testosterone concentrations. Testosterone concentrations measured before 11:00 A.M. seemed to be negatively associated with BMI and age, but this association was not significant (*p = 0.4* and *p = 0.3*, respectively). For the relationships between laboratory values (testosterone, LH, FSH and SHBG) and genotype, GH treatment, BMI, and age, see Tables S2 and S3.

### 3.4. Types of Hypogonadism

Pre-TRT LH and FSH levels were available in 33 males. Seven patients had central hypogonadism (21%), seven had primary hypogonadism (21%), but the majority had a combination of hypothalamic and testicular dysfunction (*n* = 18, 55%), Table 4.

### 3.5. Literature Review

We found 13 articles that described hypogonadism in adult males with PWS and fulfilled the inclusion criteria (Table 5 and Table 6). Most articles defined hypogonadism as a low serum testosterone concentration. The prevalence of hypogonadism ranged from 57% to 100%, with 6 of 10 articles reporting a prevalence of ≥90%. Central hypogonadism was the most common form of hypogonadism, while primary and mixed forms of hypogonadism were also reported. Multiple articles reported laboratory measurements and showed that testosterone and inhibin B values were below the reference range in most patients.

### 3.6. Expert Panel and Clinical Recommendations

**Figure 6:**
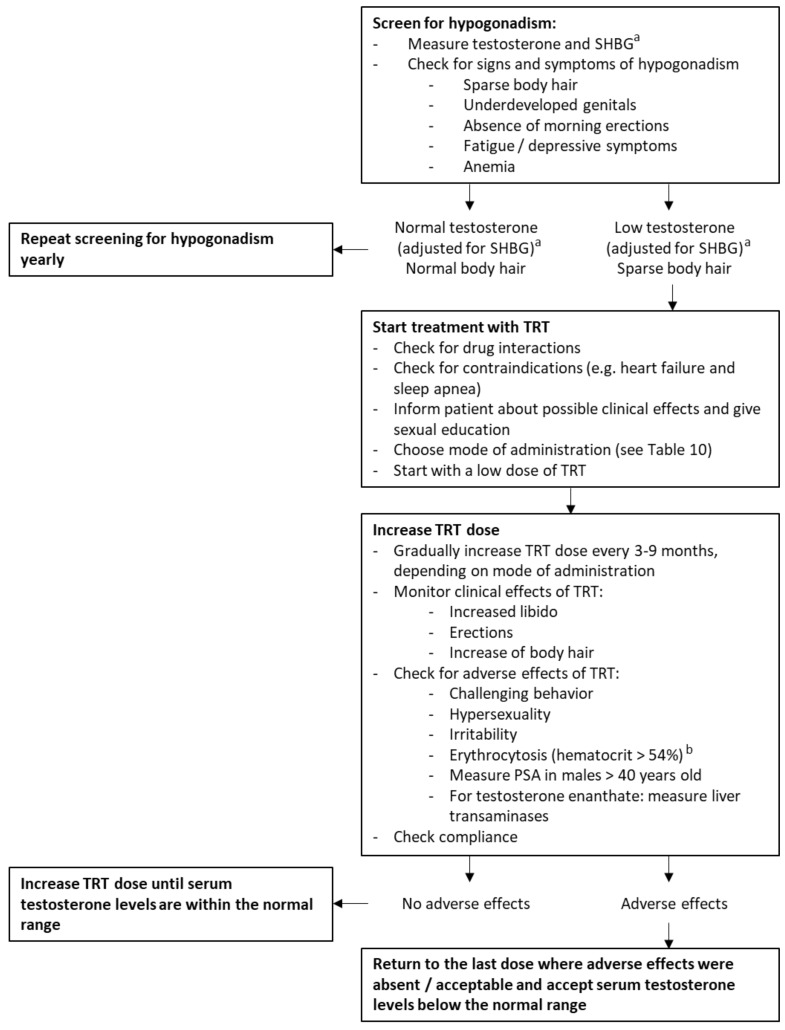
Recommendations for hypogonadism in adult males with PWS. Abbreviations: sex hormone binding globulin (SHBG), testosterone replacement therapy (TRT). ^a^ Instead of total testosterone and SHBG, free testosterone can also be measured to diagnose hypogonadism in males with PWS. ^b^ Based on the Endocrine Society Clinical Practice Guideline for testosterone therapy in men with hypogonadism [38].

Eleven experts (C.P., M.C., A.P.G., C.H., T.P.M., G.G., An.C., As.C., H.J.H., J.L.M. and L.C.G.d.G.) shared their experience with the treatment of hypogonadism in adult males with PWS (Table 7, Table 8 and Table 9). The most frequently used types of TRT were transdermal gel and short-acting injections. Starting dose and dose increase for each modality varied between experts. Additionally, one expert stated that he routinely measured estradiol concentrations in adult males with PWS, while three experts stated that they measured estradiol only in males with gynaecomastia. The other seven experts never measured estradiol in males. The advantages and disadvantages of injections and transdermal gel reported by the experts, supplemented with advantages and disadvantages mentioned in the Endocrine Society Clinical Practice Guideline for testosterone therapy in men with hypogonadism [38], are summarized in Table 10. Based on this cohort study, a review of the literature and the expert panel discussion, we have made recommendations for the screening and treatment of hypogonadism in adult males with PWS (Table 10 and Figure 6).

## 4. Discussion

Hypogonadism is present in nearly all adult males with PWS (98%). Although untreated hypogonadism was associated with obesity and decreased serum hemoglobin concentrations, adequate treatment leading to normal serum testosterone levels was only achieved in about one third of the patients.

### 4.1. Type of Hypogonadism

Although PWS is characterized by its hypothalamic dysfunction, hypogonadism in PWS can also be of testicular origin. *MKRN3, NDN*, and *SNORD116*, genes that are located in the PWS critical region, have been associated with GnRH secretion and hypothalamic dysfunction leading to hypogonadism [39]. Testicular dysfunction in men with PWS could be related to abnormal histology of the tubules and absence of spermatogonia [20,40,41]. *C15orf2*, another gene in the PWS critical region, is expressed in the testes and might play a role in spermatogenesis and therefore in the disturbance of the FSH/inhibin B axis [35,42].

The maturation of Leydig and Sertoli cells in PWS occurs independently [16]. Therefore, the LH/testosterone and FSH/inhibin B axes can be affected separately, either at the central or primary level. This leads to three forms of hypogonadism in PWS: central (low/normal LH with low testosterone and low/normal FSH with low inhibin B), primary (elevated LH with low testosterone and elevated FSH with low inhibin B), and a combination of hypothalamic and testicular dysfunction. We found a high prevalence of this mixed form of hypogonadism (55%). Central (21%) and primary hypogonadism (21%) were equally common. As previous reports show that most adult males with PWS have low inhibin B values [16,18], we assume that this is also true for our population. In that case, FSH levels in our patients might be in the normal range due to hypothalamic dysfunction of the FSH/inhibin B axis, with normal FSH levels being inadequately low for low inhibin B levels. However, as inhibin B measurements are not routinely measured as part of our standard patient care, we cannot draw any firm conclusions about the presence of hypothalamic dysfunction of the FSH/inhibin B axis.

### 4.2. Undertreatment

In one third of the patients, normal serum testosterone levels could not be achieved due to challenging behavior. Although this challenging behavior seemed related to the start of TRT or an increase in testosterone dose, it was not possible to exclude placebo effect or other factors that might aggravate challenging behavior. Eighteen patients required testosterone dose reduction due to the development of challenging behavior. In only 11 (61%) of these patients did a reduction in TRT dose reduce the challenging behaviors, suggesting that TRT may not necessarily be the cause of the increase in challenging behaviors.

Remarkably, low testosterone concentrations were still seen in patients with higher prescribed testosterone doses. This might be due to non-compliance or variability in biochemical measurements. Additionally, although SHBG concentrations were normal initially, they may have decreased over time.

### 4.3. Importance of Treatment of Hypogonadism

Untreated hypogonadism can aggravate PWS-related health issues including osteoporosis, decreased muscle mass and increased fat mass, fatigue, and impaired cardiovascular health.

#### 4.3.1. Osteoporosis

TRT increases bone mineral density in hypogonadal males without [43] and with PWS [17,44]. Therefore, it is important to treat hypogonadism to avoid osteoporosis and subsequent fractures.

#### 4.3.2. Muscle and Fat

Higher testosterone concentrations are associated with decreased fat mass and increased fat-free mass, muscle volume, and muscle strength [43,45]. A significant decrease in body fat percentage and increase in lean body mass in males with PWS has been demonstrated after two years of TRT [17]. Patients with PWS already have a decreased muscle mass and an increased fat mass, related to impaired exercise tolerance, hyperphagia and impaired GH secretion [36,46]. This abnormal body composition leads to a vicious cycle of low muscle mass, poor exercise tolerance and little physical activity, which further decreases muscle mass. Treatment of hypogonadism in males with PWS is important to increase exercise tolerance and improve muscle mass and strength to help break the vicious cycle.

After correction for age, we found that males with untreated hypogonadism had a significantly higher BMI compared to males who did not have hypogonadism or received TRT. However, patients receiving TRT are probably more likely to receive other interventions that may influence body weight, such as GH replacement, physiotherapy or dietary treatment.

#### 4.3.3. Fatigue

Treatment of non-PWS male hypogonadism can have beneficial effects on vitality and quality of life, and reduce fatigue and depressive symptoms [43,47,48,49], although studies have reported mixed results [50].

The relation between hypogonadism and fatigue may be partly explained by anemia. In the general population, treatment of male hypogonadism increases hemoglobin levels and reduces anemia [43,45]. In our population, males with untreated hypogonadism had significantly lower hemoglobin levels than those without untreated hypogonadism.

At baseline, we did not find a significant difference in subjective complaints of fatigue and daytime sleepiness between males with and without untreated hypogonadism. We did not systematically assess the psychological effects after the start of TRT. However, in our clinical experience, we did see improvements in mood and vitality in many males after the start of TRT. Further research is needed to longitudinally assess the effect of TRT on fatigue and quality of life in adult males with PWS.

#### 4.3.4. Cardiovascular Health

Cardiovascular (CV) risk factors, including obesity, hypertension, Type 2 diabetes mellitus, sleep apnea and hypercholesterolemia are prevalent in adults with PWS [36], leading to a high risk of CV disease and CV mortality at a young age [51,52]. Hypogonadism has been associated with poor CV outcomes [53,54] and TRT may improve CV health, although contradictory data have been reported and more research is needed [53,54].

### 4.4. TRT Warnings and Precautions

TRT can cause behavioral challenges, irritability, and aggressive behavior [35]. However, Kido et al. [17] found no difference in the Modifier Overt Aggression Scale (MOAS) after two years of TRT in males with PWS and did not observe challenging behaviors caused by TRT. In our population, we did see behavioral challenges during TRT. In 18 (32%) males, the testosterone dose was decreased because of behavioral challenges, leading to inadequate serum testosterone concentrations. These differences between our study and Kido et al. [17] can partly be explained by the fact that, as opposed to Kido et al., we did not exclude patients based on behavioral problems at baseline, and that Kido et al. used a different form of TRT, namely monthly intramuscular injections of 125 mg testosterone enanthate, which is half of the conventional dose. However, short-acting testosterone injection regimes (2–4 weekly) might be expected to increase the risk of behavioral problems as a result of supra-physiological testosterone concentrations shortly after injection compared to the most frequently used modality in our population, testosterone gel [38].

As TRT can induce libido and sexual activity in patients who are used to lifelong hypogonadism, it is important to inform the patients and their caregivers about these possibly confusing new feelings. A clear ‘code of conduct’ should be discussed with regard to sexual activity before starting TRT, in order to prevent inappropriate sexual behavior. It is important to ask about sexuality, sexual function, libido and erections to identify problems and to evaluate the effect of TRT. When discussing sexuality, it is important to use direct and very simple language.

A majority of the physicians of the expert panel discussion reported that normal testosterone values could be reached without causing behavioral problems in their population of adults with PWS. This could be related to the use of testosterone injections instead of transdermal gel or a slower increase in testosterone dose. Additionally, in the Dutch cohort a neuropsychologist was involved in the multidisciplinary care for adults with PWS, which could have led to greater identification of behavioral issues resulting from TRT. Further research is needed to accurately assess the differences in behavioral challenges between all treatment regimens and centers.

### 4.5. Recommendations

Based on the combined clinical experience of all co-authors, we propose clinical recommendations for the treatment of hypogonadism in adult males with PWS for TRT, see Table 10 and Figure 6. We wish to highlight issues that are especially relevant when treating hypogonadism in males with PWS. For the non-PWS specific aspects of TRT, we recommend referring to the general guidelines for the treatment of hypogonadism in men for topics not discussed here [38]. As clinical practice differed greatly among experts, we provide ranges for the possible starting dose and dose increase of TRT.

#### 4.5.1. Interpretation of Hormone Levels

Whenever possible, testosterone concentrations should be measured in the morning. When a low total testosterone concentration is found, we recommend measurement of SHBG levels before starting TRT. SHBG levels can be low due to obesity, which is often present in patients with PWS [55]. During follow-up, SHBG measurement may need to be repeated if obesity or insulin resistance develop or worsen [38]. Alternatively, free testosterone levels can be measured instead of total testosterone and SHBG.

#### 4.5.2. Sleep Apnea

Sleep apnea is common in PWS [8,56,57] and TRT can worsen symptoms of obstructive sleep apnea [57]. Therefore, we recommend screening for obstructive sleep apnea before starting TRT, and if present to treat this condition. After the start of TRT, polysomnography should be performed if clinical signs of sleep apnea develop.

#### 4.5.3. Drug Interactions

As several drugs interact with TRT, we recommend checking for possible drug interactions before starting TRT. As use of psychotropic and anti-epileptic drugs is common in adults with PWS, it is especially important to check for interactions with drugs like selective serotonin reuptake inhibitors, anti-epileptic medication and psychostimulants like modafinil [58,59,60,61,62,63]. As these drugs may influence serum testosterone concentrations, adjustment of the dose of TRT might be needed. TRT can also interact with growth hormone (GH) treatment. As TRT can increase insulin-like growth factor 1 (IGF-1) concentrations [45,64,65], it is important to evaluate and, if necessary, adjust the GH dose after initiation of TRT.

#### 4.5.4. Cardiac Failure

As the use of androgens might induce fluid retention [38,66] and cardiac problems are common in patients with PWS [57], we recommend excluding or appropriately managing heart failure before starting TRT. As patients with PWS are often unable to accurately express their cardiac symptoms due to intellectual disability and a high pain threshold [7], and leg edema is not a reliable marker of heart failure in patients with PWS [67], heart disease in adults with PWS can easily remain undiagnosed. Therefore, we recommend arranging an echocardiogram, checking serum N-terminal pro b-type natriuretic peptide (NT-proBNP) concentrations and/or consulting a cardiologist prior to the commencement of TRT in case of pitting edema or exercise-related shortness of breath. It should be noted that NT-proBNP can be false-negative in patients with obesity [68].

#### 4.5.5. Challenging Behavior

To avoid the development or worsening of aggression, hypersexuality and temper tantrums, we recommend starting with a low dose of TRT and gradually increasing the dose every 3–6 months for testosterone gel and short-acting injections and every 3–9 months for long-acting injections. If increasing the dose is impossible due to altered (sexual) behavior, we recommend returning to the last dose where behavior was still acceptable.

#### 4.5.6. Mode of Administration

Among the clinicians participating in the international expert panel, many different treatment regimens were used. Due to the need for gradual increase and the possibility for rapid dose reduction in case of behavioral challenges, our general recommendation is to use transdermal gel instead of injections when initiating TRT [69,70]. However, once established on a final transdermal TRT dose with satisfactory behavioral profile, it may be possible to switch to intramuscular injections. We advise against using oral testosterone preparations because of the risk of liver damage and increased intestinal conversion to dihydrotestosterone, preventing aromatisation to estrogen and thus hindering bone protection [38,71,72,73].

#### 4.5.7. Erythrocytosis

As long-term treatment with testosterone might generate erythrocytosis, we recommend to measure hemoglobin and hematocrit regularly during TRT, similar to the recommendations for TRT in non-PWS males [38]. When erythrocytosis occurs, TRT should be withheld until hematocrit has returned to the normal range. Then, TRT can be resumed at a lower dose [38].

#### 4.5.8. Prostate and Liver

We recommend measurement of prostate specific antigen (PSA) in men who are over 40 years old, as the long-term effects of TRT on the prostate in PWS are unknown. A urology consult should be obtained if PSA levels increase above baseline during TRT. TRT in non-PWS men does not seem to be associated with benign prostatic hyperplasia or lower urinary tract symptoms [74]. In addition, increased levels of liver transaminases may occur during treatment with testosterone enanthate and should be monitored [75,76].

#### 4.5.9. Non-Compliance

Non-compliance is frequent in adults with PWS [77], even compared to non-PWS adults with intellectual disability [78]. Although many patients are grateful to receive TRT and have no problems with adhering to their TRT regime, we found that non-compliance to TRT was often seen (certain non-compliance in 11% and a high suspicion of non-compliance in 9%), especially when the patient administered his own medication. However, as figures about non-compliance are, by definition, unreliable, actual non-compliance may be more frequent. Therefore, we recommend asking about barriers that may reduce compliance such as practical barriers (e.g., inability to administer testosterone gel, lack of caregivers who can administer the gel) and other concerns (e.g., fear of adverse events). As indicated by multiple experts during our survey, compliance might be better in patients receiving monthly or three-monthly testosterone injections, compared to testosterone gel that requires daily administration.

### 4.6. Role of PWS Reference Centers

The TRT-related challenges may cause physicians to refrain from prescribing TRT in males with PWS. However, we want to stress the importance of adequate treatment as undertreatment can have serious health consequences. PWS reference centers can be contacted for consultation or, if geographically possible, referral. If there is no PWS reference center available, we recommend the use our algorithm for treatment of hypogonadism in men with PWS (Table 10 and Figure 6).

### 4.7. Strengths and Limitations

To our knowledge, we are the first to provide a practical flowchart for the screening and treatment of hypogonadism in males with PWS [39]. Another strength of our study is the relatively large cohort, given the fact that PWS is a rare syndrome. In addition, we have provided a comprehensive literature review of male hypogonadism in adults with PWS. However, our study also has some limitations. First, there was limited overlap in age between the group of males with untreated hypogonadism and the group of males with treated or no hypogonadism, possibly leading to residual confounding. Second, due to the circadian rhythm of testosterone, we analyzed the testosterone levels drawn before 11:00 A.M. and the testosterone levels drawn after 11:00 A.M. separately [37]. As few males had morning testosterone measurements and none had fasting testosterone measurements, we had limited power to investigate which factors influenced endogenous testosterone values. Third, physical examination reports did not always include details about lipomastia or gynaecomastia. Therefore, we cannot rule out that some men had breast enlargement that was not specifically described in their medical records. In addition, we did not measure estradiol concentrations, thus we were not able to investigate the relationship between breast enlargement and estradiol. Finally, we had too few DEXA-scans available to evaluate the effect of TRT on bone mineral density, lean body mass, and fat percentage. Further research should determine the effect of TRT on these clinical effects of TRT, as they may be more important parameters to measure the effectiveness of TRT than serum testosterone measurements, although these parameters may also be influenced by GH treatment.

## 5. Conclusions

In conclusion, hypogonadism was present in nearly all males with PWS (98%) and was often a combination of hypothalamic and testicular dysfunction. Although untreated hypogonadism was associated with obesity and decreased serum hemoglobin concentrations, treatment leading to serum testosterone levels within the normal range was only achieved in one third of the patients attending our center. In order to prevent undertreatment due to behavioral challenges or other PWS-related challenges, we provide a practical algorithm for TRT in adult males with PWS.

## Figures and Tables

**Table 1:** Baseline characteristics of 57 adult males with Prader-Willi syndrome.

	Males with PWS
	*n* = 57
Age in years, median (IQR)	29 (20–40)
BMI in kg/m^2^, median (IQR)	27 (26–32)
Overweight (BMI 25–30 kg/m^2^), *n* (%)	29 (51%)
Obesity (BMI > 30 kg/m^2^), *n* (%)	17 (30%)
Genetic subtype	
Deletion	29 (51%)
mUPD ^a^	20 (35%)
ICD	2 (4%)
Unknown	6 (11%)
Growth hormone treatment	
Only during childhood	4 (7%)
Only during adulthood	1 (2%)
Both	20 (35%)
Never	32 (56%)
Current growth hormone treatment	19 (33%)
Living situation	
With family	16 (28%)
In a specialized PWS group home	8 (14%)
In a non-specialized facility	32 (56%)
Other ^b^	1 (2%)
Education level	
Secondary vocational education	2 (4%)
Pre-vocational secondary education	2 (4%)
Special education	43 (75%)
No education	1 (2%)
Unknown	9 (16%)
Relationship status	
In a relationship with sexual intercourse	2 (4%)
In a relationship without sexual intercourse	10 (18%)
Not in a relationship	37 (65%)
Unknown	8 (14%)
Cryptorchidism	36 (63%)
Of which underwent orchidopexy, *n* (% of cryptorchidism)	34 (94%)
Of which underwent orchidectomy, *n* (% of cryptorchidism)	2 (6%)
No cryptorchidism	5 (9%)
Cryptorchidism unknown	16 (28%)
Small penile length	
Yes	22 (39%)
No	11 (19%)
Unknown	24 (42%)

Abbreviations: body mass index (BMI), paternal deletion (deletion), imprinting center defect (ICD), interquartile range (IQR), maternal uniparental disomy (mUPD), Prader-Willi syndrome (PWS). Data are presented as *n* (%), unless otherwise specified. Baseline characteristics were collected during the first visit to the multidisciplinary outpatient clinic of our PWS reference center. ^a^ In 9 patients with suspected mUPD, the parents were not available for genetic testing. Therefore, mUPD is the most likely genotype, but an ICD could not be ruled out in these patients. ^b^ One patient lived alone with ambulatory care.

**Table 2:** Hypogonadism in male adults with PWS.

	Males with PWS
	*n* = 57
Hypogonadism before screening, *n* (%)	28 (49%)
Of which treated, *n* (% of hypogonadal)	24 (86%)
Type of testosterone replacement therapy before screening	
Gel, *n* (% of treated)	12 (50%)
Injections, *n* (% of treated)	10 (42%)
Short-acting, *n* (% of treated)	9 (38%)
Long-acting, *n* (% of treated)	1 (4%)
Oral, *n* (% of treated)	2 (8%)
Newly diagnosed hypogonadism, *n* (%)	28 (49%)
Hypogonadism after screening, *n* (%)	56 (98%)
Of which currently treated, *n* (% of hypogonadal)	42 (75%)
Current type of testosterone replacement therapy	
Gel, *n* (% of treated)	37 (88%)
Injections, *n* (% of treated)	3 (7%)
Short-acting, *n* (% of treated)	3 (7%)
Long-acting, *n* (% of treated)	0 (0%)
Oral, *n* (% of treated)	2 (5%)
Decrease in testosterone dose due to challenging behavior	
Yes, *n* (%)	18 (32%)
Of which had increased testosterone concentrations, *n* (% of yes) ^a^	1 (6%)
Of which had normal testosterone concentrations, *n* (% of yes) ^a^	2 (11%)
Of which had inadequate testosterone concentrations, *n* (% of yes) ^a^	15 (83%)
Of which reported a decrease in challenging behavior after the testosterone dose was decreased, *n* (% of yes)	11 (61%)
No, *n* (%)	31 (54%)
Unknown, *n* (%)	8 (14%)
Problems with compliance to testosterone replacement therapy	
Yes, *n* (%)	6 (11%)
High suspicion of non-compliance, *n* (%)	5 (9%)
No problems with compliance reported, *n* (%)	36 (63%)
Never used testosterone replacement therapy, *n* (%)	10 (18%)
Enlarged breasts	
Yes, *n* (%)	8 (14%)
Gynaecomastia, *n* (% of enlarged breasts)	1 (13%)
Lipomastia, *n* (% of enlarged breasts)	2 (25%)
Unknown, *n* (% of enlarged breasts)	5 (63%)
No or not assessed, *n* (%)	49 (86%)

Sex hormone-binding globulin (SHBG) measurements were not repeated after the start of testosterone replacement therapy, but were normal before start of testosterone replacement therapy. ^a^ Serum testosterone concentrations measured before the decrease in testosterone dose.

**Table 3:** Effect of untreated hypogonadism in adult males with PWS.

	Number of Observations	Untreated Male Hypogonadism *n* = 32	Number of Observations	Treated/No Male Hypogonadism *n* = 25	*p*-Value	*p*-Value after Correction for Age
Age, median (IQR)	32	35 (26–50)	25	23 (19–30)	0.003	NA
BMI, median (IQR)	32	29 (27–38)	25	26 (23–27)	<0.001	0.001
Overweight, *n* (%)		15 (47%)		14 (56%)		
Obesity, *n* (%)		14 (44%)		3 (12%)		
Anemia, *n* (%)	32	11 (34%)	24	4 (17%)	0.09	0.15
Hemoglobin in mmol/L, median (IQR)	32	8.2 (8.0–9.0)	24	9.3 (8.6–9.7)	0.009	0.03
Hemoglobin in g/dL, median (IQR)	32	13.2 (12.9–14.5)	24	15.0 (13.9–15.6)		
Hematocrit in L/L, median (IQR)	18	0.43 (0.42–0.45)	18	0.45 (0.43–0.48)	0.02	0.2
Subjective complaints						
Daytime sleepiness, *n* (%)	26	17 (65%)	22	7 (32%)	0.02	0.09
Fatigue, *n* (%)	25	7 (28%)	22	4 (18%)	0.4	0.5
Sexual complaints, *n* (%)	25	3 (12%)	22	2 (9%)	0.7	NA ^a^
Temper tantrums, *n* (%)	26	13 (50%)	22	11 (50%)	1	0.7

Abbreviations: body mass index (BMI), interquartile range (IQR), not available (NA). Comparison of patients who had hypogonadism, but were not treated before screening (untreated male hypogonadism) compared to patients who either already received TRT before screening (*n* = 24) or who did not have hypogonadism (*n* = 1). Subjective complaints were scored on a 5-point Likert scale. A score of 3 or higher was seen as ‘present’. Overweight is defined as a BMI between 25 and 30 kg/m^2^ and obesity as a BMI above 30 kg/m^2^. Reference ranges for hemoglobin and hematocrit were 8.6–10.5 mmol/L (13.9–16.9 g/dL) and 0.4–0.5 L/L, respectively. ^a^ Not enough events to fit the model to correct for age.

**Table 4:** LH and FSH values in males with PWS.

		**FSH/Inhibin B Axis**		
		Hypothalamic dysfunction (low FSH) 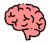	Hypothalamic or no dysfunction ^a^ (normal FSH) 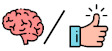	Testicular dysfunction (high FSH) 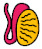
LH/Testosterone axis	Hypothalamic dysfunction (low/normal LH with low T) 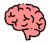	1 (3%)	6 (18%)	18 (55%)
	No dysfunction (normal LH with normal T) 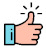	0 (0%)	0 (0%)	1 (3%)
	Testicular dysfunction (high LH with low T) 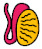	0 (0%)	0 (0%)	7 (21%)

Abbreviations: follicle stimulating hormone (FSH), luteinizing hormone (LH), testosterone (T). Laboratory measurements of LH, FSH and testosterone were available for 33 adult males. In the other 24 males no laboratory measurements were available or only laboratory measurements during testosterone replacement therapy were available. This figure has been designed using resources from Flaticon.com (from freepik and smashicons), accessed 1 June 2021. ^a^ As no inhibin B measurements were available, we did not know whether patients with normal FSH had normal function of the FSH/inhibin B axis or hypothalamic FSH/inhibin B axis dysfunction with inappropriately low FSH for the low inhibin B values. Based on previous research, we assumed that inhibin B levels were low in most adult males with PWS [16,18].

**Table 5:** Literature review hypogonadism in male adults with PWS (Part 1).

Article	*n*	Country	Age Range (Years)	Genotype (Deletion/mUPD/ICD/Translocation)	Mean BMI (kg/m^2^)	Assays Used	Definition Hypogonadism
Partsch et al. (2000) [8]	7	Germany	18–34 ^a^	All deletion or mUPD	46 ^a^	commercially available immunoassays	testosterone levels below the normal range
Whittington et al. (2002) [9] ^b^	31	United Kingdom	18–46	NA ^c^	NA	NA	undescended testes at birth and/or small genitalia
Grugni et al. (2003) [10]	7	Italy	19–29	7/0/0/0	37	FSH, LH: immunochemiluminescent assays testosterone: chemiluminescent immunoassay	testosterone levels below the normal range
Höybye et al. (2005) [11] ^b^	7	Sweden	19–36	0/1/0/0 (6 NA) ^c^	Median 28	commercially available immunoassays	low testosterone or treatment with sex steroids
Miller et al. (2008) [12] ^b^	10	Florida, USA	18–34	6/4/0/0	38	commercially available radioimmunoassays	Hypogonadotropic hypogonadism: delayed onset of puberty (i.e., >14 years) in addition to low gonadotropin levels for age
Brandau et al. (2008) [13] ^b^	20	Missouri, USA	18–45	12/8/0/0	35	FSH, LH: chemiluminescence assays testosterone: radioimmunoassay	testosterone levels below the normal range
Sode-Carlsen et al. (2010) [14] ^b^	21	Denmark, Norway, Sweden	18–39	13/1/1/0 (6 NA) ^c^	Median 25	commercially available immunoassays	low testosterone or treatment with sex steroids
Van Nieuwpoort et al. (2011) [15]	4	The Netherlands	21–42	14/1/0/0 ^a^	29	commercially available immunoassays	low testosterone or treatment with sex steroids
Radicioni et al. (2012) [16] ^b^	17	Italy	18–42	13/4/0/0	42	FSH, LH, testosterone: chemiluminescent microparticle immunoassay Inhibin B: enzymatically amplified two-site two-step sandwich-type immunoassay (ELISA) SHBG: immunoradiometric assay	testosterone and/or inhibin B levels below the normal range
Kido et al. (2013) [17]	16	Japan	18–48	15/1/0/0	33	NA	testosterone level <300 ng/dL and Tanner stage less than IV.
Hirsch et al. (2015) [18] ^b^	18	Israel	18–36	11/7/0/0	29	LH, FSH, testosterone: immunoassays Inhibin B, AMH: Two-site enzyme-linked immunosorbent assay (ELISA) SHBG: immunochemiluminescence	NA
Coupaye et al. (2016) [19] ^b^	31	France	18–58 ^a^	42/24/0/0 ^a,d^	39 ^a^	routine techniques	testosterone level <320 ng/dL or treatment with sex steroids
Matsuyama et al. (2019) [20]	11	Japan	18–NA (Mean ± SD: 19.9 ± 2.3)	6/0/1/0 (4 NA)	NA	LH, FSH: two-site enzyme immune-assay testosterone: chemiluminescent immunoassay	NA

Abbreviations: anti-müllerian hormone (AMH), body mass index (BMI), paternal deletion (deletion), follicle stimulating hormone (FSH), imprinting center defect (ICD), luteinizing hormone (LH), maternal uniparental disomy (mUPD), not available (NA), standard deviation (SD), sex hormone binding globulin (SHBG), United States of America (USA). Only articles reporting separate outcomes on adults (older than 18 years) were included. When this was not available, we contacted the authors to retrieve this information. ^a^ Data for all males and females included in this study. ^b^ Additional data was provided by the authors of this article. ^c^ All methylation positive. ^d^ Only patients with a deletion or an mUPD were included according to the inclusion criteria of this study.

**Table 6:** Literature review hypogonadism in male adults with PWS (Part 2).

Article	Hypogonadism *n* (%)	Primary Hypogonadism/Central Hypogonadism	FSH, Mean (Range)	LH, Mean (Range)	Testosterone, Mean (Range)	SHBG, Mean (Range)	Inhibin B, Mean (Range)	AMH, Mean (Range)
Partsch et al. (2000) [8]	7 (100%)	- ^a^	-	-	-	-	-	-
Whittington et al. (2002) [9]	30 (100%) (1 NA)	-	-	-	-	-	-	-
Grugni et al. (2003) [10]	5 (71%)	-	12.4 (0.1–30.6) IU/L	6.0 (0.7–15.1) IU/L	3.0 (0.5–6.9) ng/mL *10.4 (1.7–23.9) nmol/L*	-	-	-
Höybye et al. (2005) [11]	4 (57%)	0/3 (1 NA)	4.2 (2.7–10) IU/L	2.8 (0.6–5.1) IU/L	9.7 (1.9–37) nmol/L	-	-	-
Miller et al. (2008) [12]	10 (100%)	0/10	-	-	-	-	-	-
Brandau et al. (2008) [13]	17 (89%) (1 NA)	-	14.8 (0.1–52.0) IU/L	3.1 (0.1–8.0) IU/L	1.3 (0.3–4.0) ng/mL *4.5 (1.0–13.9) nmol/L*	-	-	-
Sode-Carlsen et al. (2010) [14]	14 (67%)	8/2 (4 NA)	18.5 (<0.2–64) IU/L	3.5 (<1.0–13.5) IU/L	10 (1.9–39.5) nmol/L	-	-	-
Van Nieuwpoort et al. (2011) [15]	4 (100%)	0/2 (2 NA)	Median 1.1 IU/L	Median 0.43 IU/L	Median 3.2 nmol/L	Median 17.9 nmol/L	-	-
Radicioni et al. (2012) [16]	17 (100%)	2/9 Combined: 6	11.6 (0.05–46.6) IU/L	2.5 (0.04–7.2) IU/L	3.7 (1.4–13.7) nmol/L	22.9 (6.8–42.7) nmol/L	14.0 (3.0–38.3) pg/mL	-
Kido et al. (2013) [17]	- ^b^	0/3 (13 NA)	18.9 (<0.5–43.3) IU/L	4.0 (<0.5–12.8) IU/L	99 (24–190) ng/dL *3.4 (0.8–6.6) nmol/L*	-	-	-
Hirsch et al. (2015) [18]	-	-	16.3 (0.1–55.9) IU/L	3.0 (0.1–10.5) IU/L	1.8 (0.2–4.7) nmol/L	34.2 (9.0–73.8) nmol/L	72.4 (0.1–269.0) pg/mL (*n* = 17)	12.13 (0.17–62.40) ng/mL (*n* = 16)
Coupaye et al. (2016) [19]	30 (97%)	-	Mean ± SD 13.2 ± 16 IU/L	Mean ± SD 3.2 ± 3.1 IU/L	1.3 (0.2–4.0) ng/mL *4.5 (0.7–14) nmol/L*	Mean ± SD 30.0 ± 20.0 nmol/L	Mean ± SD 36 ± 38 pg/mL	Mean ± SD 9.5 ± 15.3 ng/mL
Matsuyama et al. (2019) [20]	-	-	19.5 (7.5–30.8) IU/L	4.0 (1.0–5.3) IU/L	248 (102–509) ng/dL *8.6 (3.5–17.6) nmol/L*	-	-	-

Abbreviations: anti-müllerian hormone (AMH), follicle stimulating hormone (FSH), luteinizing hormone (LH), not available (NA), sex hormone-binding globulin (SHBG). When only laboratory measurements in non-SI units were reported, we added the converted values in *italics*. Only values for FSH, LH, and testosterone in patients who did not use sex steroid replacement therapy during blood withdrawal are included. Values that were below the measuring threshold were considered equal to the measuring threshold to calculate the mean. For example, when FSH was reported as <0.5, this was considered 0.5. ^a^ Gonadotropin levels were subnormal in all but one patient (of the population of 7 males and 12 females) and showed a reduced responsiveness to stimulation with exogenous gonadotropin-releasing hormone. ^b^ Only males with PWS with hypogonadism were included according to the in- and exclusion criteria of this study.

**Table 7:** Expert panel discussion (Part 1).

	Expert 1 and Expert 2 ^a^	Expert 3	Expert 4
(Past) experience	Short-acting injections	Long-acting injections, transdermal gel	Long-acting injections, transdermal gel
Preferred mode of administration in PWS	Short-acting injections	Transdermal gel followed by long-acting injections	Long-acting injections, transdermal gel
Mode of administration advised against	Oral testosterone	Short-acting injections, oral testosterone	None
Preferred starting dose in testosterone naïve patients	Short-acting injections: 125 mg every 3–4 weeks	Long-acting injections: 200 mg every 12 weeks Transdermal gel: 10 mg daily	Long-acting injections: 250–500 mg for the first injection Transdermal gel: 10–30 mg depending on testosterone level
Preferred follow-up dose	Short-acting injections: After 6 months: increase to 200 mg every 3–4 weeks and then 250 mg every 3–4 weeks depending on serum testosterone concentrations, clinical signs and adverse effects	Long-acting injections: Every 6–9 months: increase by 200–300 mg, depending on serum testosterone and SHBG concentration Transdermal gel: Every 3–6 months: increase by 10 mg	Long-acting injections: Depending on the increase in serum testosterone concentration, injection of 500–1000 mg after 6 weeks. After 12 weeks, depending on the testosterone level obtained, injection of 500–1000 mg, which is then continued every 12 weeks aiming for a serum testosterone concentration within the normal range Transdermal gel: Gradual increase over 1–4 weeks to 30–50 mg daily, aiming for a serum testosterone concentration within the normal range
Biochemical follow up	Testosterone, Hb, Ht after each change of TRT dose. Once the final dose of TRT has been obtained, measurement of testosterone, Hb, Ht every year	Testosterone, Hb, Ht, SHBG, estradiol every 6 months or prior to dose increase	Testosterone, LH, FSH, Hb, Ht every 6–12 months, cholesterol every 12 months
Considerations:	Only short-acting injections are reimbursed in these experts’ country (France), while transdermal gel is no longer available	Start with transdermal gel as this allows gradual dose up-titration and immediate cessation if behavioral problems occur. Once established on final transdermal dose, switch to long-acting injections as this has smoother pharmacokinetics than short-acting injections and does not need to be applied daily, though depending on patient preference may continue transdermal gel	Short-acting injections not available in the expert’s country (Sweden). Decision for long-acting injections or transdermal gel based on patient preference, most patients prefer injections instead of transdermal gel
Additional remarks		With gradual increases in testosterone dose behavioral problems do not appear to be an issue. Once established on long-acting injections measure testosterone concentrations ~1–2 months after injection and at trough just prior to injection as may need to increase injection frequency rather than dose	

Abbreviations: follicle stimulating hormone (FSH), hemoglobin (Hb), hematocrit (Ht), luteinizing hormone (LH), Prader-Willi syndrome (PWS), sex hormone-binding globulin (SHBG), testosterone replacement therapy (TRT). In this table the general considerations are described regarding testosterone replacement therapy for adult males with PWS who have not used testosterone replacement therapy before. However, based on patient preference, another treatment modality or dose could be prescribed. We defined short-acting injections as injections that have to be administered every 1–6 weeks, and long-acting injections as injections that have to be administered every 12 weeks. For this expert discussion we focused on the use of short-acting and long-acting injections, transdermal gel and oral testosterone only. Biochemical follow-up refers to the biochemical measurements performed during the titration of TRT dose. Physicians may perform additional measurements before the initiation of TRT (e.g., LH, FSH and/or SHBG to confirm the diagnosis hypogonadism) or during long-term follow-up (e.g., yearly measurement of prostate specific antigen in older men) and may change the frequency of biochemical measurement after reaching the final TRT dose. ^a^ As Expert 1 and Expert 2 worked closely together in the same PWS reference center and had the exact same clinical practice, they were combined into one column.

**Table 8:** Expert panel discussion (Part 2).

	Expert 5	Expert 6	Expert 7	Expert 8
(Past) experience	Long-acting injections, transdermal gel, oral testosterone	Short-acting injections, transdermal gel	Short-acting injections, transdermal gel	Short-acting injections, transdermal gel
Preferred mode of administration in PWS	Transdermal gel	Short-acting injections, transdermal gel	Short-acting injections, transdermal gel	Long-acting injections
Mode of administration advised against	Short-acting injections, oral testosterone	Long-acting injections, oral testosterone	Oral testosterone	None
Preferred starting dose in testosterone naïve patients	Transdermal gel: 12.5 mg daily	Short-acting injections: 50 mg every month Transdermal gel: 10 mg daily	Short-acting injections: 100–125 mg every 4 weeks Long-acting injections: 250–500 mg every 3 months Transdermal gel: 10 mg daily	Short-acting injections: 100 mg every month Transdermal gel: 50 mg daily
Preferred follow-up dose	Long-acting injections: Every 3–6 months: adjust dosing interval based on trough testosterone level Transdermal gel: Every 3–4 months: increase dose by 12.5 mg based on serum testosterone concentration and clinical response	Short-acting injections: Every 3–6 months: increase by 50 mg Transdermal gel: Every 4 weeks: increase by 10 mg	Short-acting and long-acting injections: Low dose for many years Transdermal gel: Every 6–8 weeks: increase by 10 mg	Short-acting injections and transdermal gel: Check up to see if testosterone has normalized after 3–4 months and increase dose based on serum testosterone concentration and clinical response
Biochemical follow up	Testosterone, Hb, Ht every 6–12 months	LH, FSH, testosterone, Hb, Ht, liver transaminases every 6 months and prior to any dose modification	Testosterone, Hb, Ht, liver transaminases every 6–8 months	Testosterone, Hb, Ht every 3–4 months
Considerations	Start with transdermal gel as this can be stopped quickly and is most suitable due to more physiological testosterone concentrations. When patient achieves normal testosterone level, either continue transdermal gel or switch to long-acting injections, based on patient preference.	Transdermal gel when the patient is compliant and the family reliable, otherwise short-acting injections.	Transdermal gel is most suitable as it results in more physiological testosterone concentrations and can be stopped instantly if behavioral problems appear, but compliance is better with injections.	Long-acting would be most suitable, but is not reimbursed in this country (Spain).
Additional remarks				

Abbreviations: follicle stimulating hormone (FSH), hemoglobin (Hb), hematocrit (Ht), luteinizing hormone (LH), Prader-Willi syndrome (PWS), sex hormone-binding globulin (SHBG), testosterone replacement therapy (TRT). In this table the general considerations are described regarding testosterone replacement therapy for adult males with PWS who have not used testosterone replacement therapy before. However, based on patient preference, another treatment modality or dose could be prescribed. We defined short-acting injections as injections that have to be administered every 1–6 weeks, and long-acting injections as injections that have to be administered every 12 weeks. For this expert discussion we focused on the use of short-acting and long-acting injections, transdermal gel and oral testosterone only. Biochemical follow-up refers to the biochemical measurements performed during the titration of TRT dose. Physicians may perform additional measurements before the initiation of TRT (e.g., LH, FSH and/or SHBG to confirm the diagnosis hypogonadism) or during long-term follow-up (e.g., yearly measurement of prostate specific antigen in older men) and may change the frequency of biochemical measurement after reaching the final TRT dose.

**Table 9:** Expert panel discussion (Part 3).

	Expert 9	Expert 10	Expert 11
(Past) experience	Short-acting injections, transdermal gel	Short-acting injections, long-acting injections, transdermal gel	Transdermal gel, short-acting injections, long-acting injections, oral testosterone
Preferred mode of administration in PWS	Transdermal gel	Short-acting injections, transdermal gel	Transdermal gel
Mode of administration advised against	Oral testosterone	None	Oral testosterone
Preferred starting dose in testosterone naïve patients	Short-acting injections: 50–100 mg, depending on serum testosterone concentration and age Transdermal gel: 12.5–25 mg, depending on serum testosterone concentration and age	Short-acting injections: 25 mg every week or 50 mg every month Transdermal gel: 25 mg daily	Transdermal gel: 10 mg daily
Preferred follow-up dose	Short-acting injections: Every 6 months: increase by 50 to 100 mg Transdermal gel: Every 6 months: increase by 12.5 to 25 mg	Short-acting injections: Every 3 months: increase by 25 mg Transdermal gel: Every 3 months: increase by 25 mg	Transdermal gel: Every 4 weeks: increase by 10 mg
Biochemical follow up	Testosterone, Hb, Ht every 6 months	Testosterone, Hb, Ht, inhibin B every 3 months	LH, FSH, testosterone, Hb, Ht every 3 months
Considerations	Transdermal gel is most suitable as this results in stable serum testosterone concentrations, but this requires a reliable caregiver and can cause skin irritation and skin picking.	Short-acting injections and gel are tolerated best and easy to dose adjust.	Transdermal gel is most suitable as it can be stopped immediately when behavioral challenges occur due to the short half-life time.
Additional remarks			Use gel in the morning and apply gel to shoulders, not belly (due to increased abdominal fat in PWS).

Abbreviations: follicle stimulating hormone (FSH), hemoglobin (Hb), hematocrit (Ht), luteinizing hormone (LH), Prader-Willi syndrome (PWS), sex hormone-binding globulin (SHBG), testosterone replacement therapy (TRT). In this table the general considerations are described regarding testosterone replacement therapy for adult males with PWS who have not used testosterone replacement therapy before. However, based on patient preference, another treatment modality or dose could be prescribed. We defined short-acting injections as injections that have to be administered every 1–6 weeks, and long-acting injections as injections that have to be administered every 12 weeks. For this expert discussion we focused on the use of short-acting and long-acting injections, transdermal gel and oral testosterone only. Biochemical follow-up refers to the biochemical measurements performed during the titration of TRT dose. Physicians may perform additional measurements before the initiation of TRT (e.g., LH, FSH and/or SHBG to confirm the diagnosis hypogonadism) or during long-term follow-up (e.g., yearly measurement of prostate specific antigen in older men) and may change the frequency of biochemical measurement after reaching the final TRT dose.

**Table 10:** Recommendations for different testosterone formulations.

TRT Formulation	Examples	Starting Dose	Dose Increase	Advantages	Disadvantages
Transdermal gel	Testosterone (e.g., Androgel^®^, Testim^®^, Tostran^®^, Testogel^®^, Testavan^®^)	10 mg daily	Increase dose by 10 mg every 3–6 months until testosterone values within the normal range are achieved	- Flexible dosing - Easy application - Metered dosing with certain brands allows easy dose titration - Good skin tolerability - Less erythrocytosis compared to injections - Less fluctuation of serum testosterone concentrations than testosterone enanthate or cypionate	- Potential of transfer to a female partner or child by direct skin-to-skin contact - Testosterone concentrations may vary between applications - Skin irritation in a small proportion - Moderately high dihydrotestosterone concentrations (of unknown significance) - Some patients may have difficulties administrating the gel - Has to be administered every day, which may lead to non-compliance
Short-acting injections	Testosterone decanoate/isocaproate/phenylpropionate/propionate mixture (e.g., Sustanon^®^) Testosterone enanthate or cypionate (e.g., Xyosted^®^, Andortardyl^®^)	50–125 mg every month	Increase dose by 25–100 mg every 3–6 months until serum testosterone concentrations within the normal range are achieved	- Relatively inexpensive if self-administered - Flexible dosing	- Requires intramuscular injections - Risk of high peak testosterone concentrations shortly after injection - Variation in serum testosterone concentrations may be associated with fluctuations in symptoms - Coughing episode reported immediately after injection in a small number of men
Long-acting injections	Testosterone undecanoate (e.g., Nebido^®^, Reandron^®^, AVEED^®^)	200–500 mg every 3 months	Increase dose by 200–500 mg every 3–9 months until serum testosterone concentrations within the normal range are achieved	- Infrequent administration - Serum testosterone concentrations are maintained in the normal range in most treated men - Stable serum testosterone concentrations between injections	- Requires intramuscular injection of a large volume (3 or 4 mL) - Need to be administered by primary care nurse or physician - Coughing episode reported immediately after injection in a small number of men - Higher chance of erythrocytosis compared to gel

Recommendations for the treatment of hypogonadism in adult males with PWS who have not used testosterone replacement therapy before. Advantages and disadvantages are based on the expert panel discussion and the Endocrine Society Clinical Practice Guideline for testosterone therapy in men with hypogonadism [38].

## Data Availability

The datasets generated during and/or analyzed during the current study are not publicly available but are available from the corresponding author on reasonable request.

## Supplementary Materials

The following are available online at https://www.mdpi.com/article/10.3390/jcm10194361/s1, Table S1: full search strategy (Embase), Table S2: laboratory values in adult males with PWS (Part 1), Table S3: laboratory values in adult males with PWS (Part 2).
