# Supplementary material for: Hypogonadism in Adult Males with Prader-Willi Syndrome—Clinical Recommendations Based on a Dutch Cohort Study, Review of the Literature and an International Expert Panel Discussion
Source: J Clin Med. 2021 Sep 24;10(19):4361. doi: 10.3390/jcm10194361 (PMC8509256; doi:10.3390/jcm10194361)
Supplement: Supplementary file 1 [file jcm-10-04361-s001.zip › jcm-1350518-supplementary.pdf]

Supplementary Materials

Table S1. Full search strategy (Embase)

('Prader Willi syndrome'/exp OR 'mkrn3 gene'/de OR 'makorin ring finger protein 3'/de OR 'magel2 gene'/de OR 'magel2 protein'/de OR 'necdin'/de OR 'small nuclear ribonucleoprotein polypeptide n'/de OR 'snrpn gene'/de OR 'snrpn protein'/de OR 'e6 associated protein'/de OR ((Prader* NEAR/3 Willi*) OR PraderWilli OR mkrn3 OR makorin-3 OR znf127 OR D15S9 OR RNF63 OR EC-2.3.2.27 OR ZFP127 OR CPPB2 OR magel2 OR magel-2 OR ((makorin) NEAR/3 (ring) NEAR/3 (3)) OR ((zinc) NEAR/3 (finger) NEAR/3 (127)) OR ((ring) NEAR/3 (finger) NEAR/3 (63)) OR ((MAGE OR melanoma) NEAR/3 (family) NEAR/3 (L2)) OR ((necdin-like OR MAGE-like) NEAR/3 (protein) NEAR/3 (1 OR 2)) OR NDNL1 OR MAGE-Like-2 OR SHFYNG OR PWLS OR NM15 OR (NDN NOT nonclassic-differentiation-number*) OR necdin* OR HsT16328 OR PWCR OR C15orf2 OR NPAP1 OR NPAP-1 OR Nuclear-Pore-Associated-Protein-1 OR ((chromosome-15) NEAR/3 (open-reading-frame* OR ORF*) NEAR/3 (2)) OR SNURF-SNRPN* OR SNRPN* OR Small-Nuclear-Ribonucleoprotein-Polypeptide-N* OR Small-Nuclear-Ribonucleoprotein-Associated-Protein-N* OR Tissue-Specific-Splicing-Protein* OR Sm-Protein-D OR SM-Protein-N OR HCERN3 OR (((Sm-N OR SMN OR Sm-D OR PET1 OR BEY OR PED) AND (gene OR genes OR genetic OR genome* OR genomic)) NOT (sec*-malign*-neoplas* OR survival-motor-neuro*)) OR Small-Nucl*-Ribonucleoprot*-N OR RT-LI OR PWCR OR SNORD107 OR SNORD-107 OR (("C/D" OR CD) NEAR/3 (box*) NEAR/3 (107 OR 64 OR 109A OR 116 OR 115 OR 109B)) OR HBII-436 OR RF01164 OR SNORD64 OR HBII-13 OR RF00570 OR SNORD109A OR HBII-438A OR RF01278 OR SNORD116 OR HBII-85 OR PWCR1 OR SNORD115 OR HBII-52 OR RNHBII52 OR RF00105 OR SNORD109B OR HBII-438B OR RF01278 OR KIAA1899):ab,ti,kw) AND ('gonadal disease'/exp OR 'steroid receptor'/exp OR 'bone characteristics and functions'/exp OR 'sex hormone binding globulin'/de OR 'luteinizing hormone derivative'/exp OR 'follitropin'/exp OR 'inhibin'/exp OR 'infertility'/exp OR 'sexual dysfunction'/exp OR 'sex'/exp OR 'gonadorelin derivative'/exp OR 'gonadorelin receptor'/de OR 'luteinizing hormone receptor'/de OR 'menstruation disorder'/exp OR 'menstrual cycle'/exp OR 'birth control'/de OR 'contraception'/exp OR 'safe sex'/exp OR 'contraceptive device'/exp OR 'genital system'/exp OR 'steroid'/exp OR 'Muellerian inhibiting factor'/de OR 'menopause and climacterium'/exp OR 'ovary polycystic disease'/de OR 'wolffian duct'/de OR 'sexual characteristics'/exp OR 'breast'/exp OR 'breast development'/de OR 'endocrine breast disease'/exp OR 'breast malformation'/exp OR 'hair'/exp OR 'genital system function'/exp OR 'pregnant woman'/de OR 'reproduction'/de OR 'sexual dysfunction'/exp OR 'osteoporosis'/exp OR 'osteopenia'/exp OR 'bone characteristics and functions'/exp OR 'fracture'/exp OR 'aggression'/exp OR 'dual energy X ray absorptiometry'/de OR 'problem behavior'/de OR 'behavior disorder'/de OR 'hypersexuality'/exp OR 'genital system disease'/exp OR 'genital malformation'/exp OR 'orchidopexy'/de OR 'spontaneous abortion'/de OR 'prolactin derivative'/exp OR 'precocious puberty'/exp OR 'alpha fetoprotein'/de OR 'bone maturation'/de OR 'aromatization'/de OR 'aromatase deficiency'/de OR 'aromatase'/de OR 'cholesterol monooxygenase (side chain cleaving)'/de OR 'steroid 17alpha monooxygenase'/de OR '3(or 17)beta hydroxysteroid dehydrogenase'/de OR (gonad* OR hypogonad* OR hypogenital* OR hypergonad* OR eugonad* OR normogonad* OR hypergenital* OR androgen* OR estrone OR estriol OR trihydroxyestrin* OR estrogen* OR estradiol* OR androst* OR dihydrotestosteron* OR dehydroepiandrosteron* OR prasteron* OR dehydroisoandrosteron* OR testosteron* OR hydroxypregnenolone* OR pregnenolone* OR progesteron* OR progestin* OR SHBG OR sex-hormone-binding-globulin* OR LH OR luteinizing-hormone* OR luteinising-hormone* OR lutropin* OR FSH OR follitropin* OR follicle-stimulating-hormone* OR inhibin* OR ((pregnan*) NEAR/3 (woman OR women) NEAR/3 (with-IDD OR with-ID OR with-Prader)) OR fertil* OR infertil* OR infecund* OR sex OR sexual* OR orgasm* OR transsexual* OR bisexual* OR heterosexual* OR homosexual* OR gay OR straight OR lesbian* OR GnRH OR choriogonadotropin-receptor* OR amenorrhea* OR dysmenorrhea* OR menorrhagia* OR metrorrhagia* OR oligomenorrhea* OR menstrual* OR premenstrual* OR menstruat* OR contracept* OR condom* OR birth-control* OR IUD OR intrauterine-device* OR depo-provera* OR ovulat* OR ovar* OR testis OR testes OR testicular* OR ((granulosa* OR follicular* OR theca* OR sertoli* OR Leydig* OR luteal* OR cumulus*) NEAR/3 (cell*)) OR follic* OR phallic* OR Müllerian* OR Muellerian* OR fallopian* OR cervix OR endocervix OR cervical OR uterus OR uterine OR vagina* OR penis* OR penile OR phallus OR prostate* OR scrotum* OR sperm* OR steroid* OR menopaus* OR premenopaus* OR perimenopaus* OR postmenopause* OR climacterium* OR galactorrhea* OR PCOS OR ((polycystic*) NEAR/3 (ovar*) NEAR/3 (syndrome* OR disease*)) OR ((Stein) NEXT/3 (Leventhal)) OR ((wolff* OR archinephric* OR mesoneph*) NEAR/3 (duct*)) OR puberty OR pseudopuberty OR pubescence OR genital* OR breast* OR mamma* OR nipple* OR thelarche* OR hair* OR beard* OR gynecomast* OR gynaecomast* OR ejaculat* OR erection* OR erectile* OR sperm* OR libido* OR orgasm* OR anorgasm* OR osteoporo* OR ((bone*) NEAR/3 (densit* OR mass* OR health* OR strength* OR matur*)) OR fractur* OR aggress* OR DEXA OR DXA OR ((dual-energy) NEAR/3 (x-ray OR xray OR roentgen-ray)) OR ((problem* OR challeng*) NEAR/3 (behavior* OR behaviour*)) OR hypersexual* OR hyposexual* OR micropenis* OR orchidopex* OR orchiopex* OR ((luteal* OR secretor* OR premenstrual* OR proliferative*) NEXT/1 (phase*)) OR endomet* OR corpus-luteum* OR menarche* OR

miscarriage* OR spontane*-abortion* OR prolactin* OR semiferous* OR andropause* OR virilization* OR androstane* OR phallic* OR ((seminal* OR semen*) NEAR/3 (vesic*)) OR ((male* OR man OR men OR boy*) NEAR/3 (feminization* OR feminisation*)) OR ((female* OR woman OR women OR girl*) NEAR/3 (masculinization* OR masculinisation*)) OR alpha-fetoprotein* OR pubarche* OR clitoris* OR 5-alpha-reductase* OR 5alpha-reductase* OR aromat* OR androstanedio* OR ((cholesterol*) NEAR/3 (side-chain-cleav* OR monooxygenase* OR desmolase*)) OR CYP11A1 OR P450scc OR ((17α OR 17 OR 17alpha) NEAR/3 (hydroxylase* OR monooxygenase* OR desmolase*)) OR 17-20-lyase OR ((3β OR 17β OR 3beta OR 17beta OR 3-beta OR 17-beta) NEXT/1 (hydroxysteroid) NEXT/3 (dehydrogenase* OR oxidoreductase*)) OR 3β-HSD OR 17β-HSD OR 3beta-HSD OR 17beta-HSD OR 3-beta-HSD OR 17-beta-HSD OR reproduction* OR reproductive*):ab,ti,kw) OR (('Prader Willi syndrome'/exp OR ((Prader* NEAR/3 Willi*) OR PraderWilli):ab,ti,kw) AND ('BRCA1 protein'/de OR 'BRCA2 protein'/de OR (BRCA1 OR BRCA2):ab,ti,kw)) NOT ([Conference Abstract]/lim AND [1800-2017]/py) AND [English]/lim

The search strategy was adjusted, but similar search terms were used for the databases Medline (Ovid), Web of Science Core Collection, and the Cochrane Central Register of Controlled Trials. Additionally, references were searched. The search term was used to identify papers describing hypogonadism in both males and females with PWS. For this manuscript, articles that only described hypogonadism in females with PWS were excluded manually.

Table S2. Laboratory values in adult males with PWS (Part 1).

	Reference Range for Adult Males	*n*	Total *n* = 57	Deletion *n* = 29	mUPD *n* = 20	*p*-Value	Current GH treatment *n* = 19	No current GH treatment *n* = 38	*p*-Value	*p*-Value After Correction for Age
Testosterone (nmol/L)										
Before 11:00 AM		14	5.6 (3.2 – 7.4)	6.2 (3.3 – 10.5)	4.2 (0.9 – 6.3)	0.3	6.2 (4.1 – 11.4)	3.7 (2.2 – 6.5)	0.4	NA [b]
After 11:00 AM	10.0 – 30.0	16	2.0 (0.8 – 4.9)	1.5 (0.8 – 4.7)	2.1 (0.8 – 5.2)	0.9	NA [a]	2.1 (0.8 – 4.9)	NA	NA
LH (IU/L)										
Before 01-02-2019	1.5 – 8.0	30	3.3 (1.2 – 8.1)	2.0 (0.7 – 6.1)	3.9 (1.2 – 7.8)	0.7	5.4 (2.3 – 7.7)	2.4 (0.9 – 8.4)	0.6	0.06
After 01-02-2019	1.0 – 5.5	3	1.9, 2.8, 11.0 [c]	1.9 [c]	11.0 [c]		1.9, 2.8 [c]	11.0 [c]		
FSH (IU/L)										
Before 01-02-2019	2.0 – 7.0	31	13.5 (6.4 – 34.5)	11.6 (5.4 – 30.5)	16.8 (5.8 – 46.2)	0.2	20.8 (9.8 – 44.1)	13.3 (5.0 – 34.5)	0.6	0.1
After 01-02-2019	0.8 – 5.1	3	4.8, 7.2 & 58.0 [c]	4.8 [c]	58.0 [c]		4.8, 7.2 [c]	58.0 [c]		
SHBG (nmol/L)	10.0 – 70.0	28	29.9 (20.3 – 49.4)	23.8 (19.8 – 44.2)	35.5 (23.8 – 57.8)	0.2	15.5 (8.1 – 19.4)	35.5 (23.7 – 55.5)	0.003	0.2

Abbreviations: growth hormone (GH), follicle stimulating hormone (FSH), luteinizing hormone (LH), maternal uniparental disomy (mUPD), paternal deletion (deletion), sex hormone binding globulin (SHBG), number of observations (*n*). Data are presented as median values (IQR). Laboratory values for patients at baseline or during the last measurement available before the start of testosterone replacement therapy for LH, FSH, and testosterone. For SHBG the measurement closest to baseline was used. Values that were below the measuring threshold were considered equal to the measuring threshold to calculate the median and IQR. For example, when FSH was below 0.5, this was considered 0.5. For LH and FSH, measurements before and after the change in measuring method on 01-02-2019 are calibrated differently, therefore these measurements are described separately and a variable indicating whether the values were measured before or after 01-02-2019 was added to the model to calculate the *p*-value. [a] There were no observations in this group, all patients had either a testosterone measurement before 11:00 AM, or no testosterone measurement before testosterone replacement therapy available. [b] Not enough testosterone measurements to fit the model to correct for age. [c] Individual measurements are given as there were too few observations to calculate a median and IQR.

Table S3. Laboratory values in adult males with PWS (Part 2).

	n	**BMI < 25** *n* **= 11**	**BMI 25 – 30** *n* **= 29**	**BMI > 30** *n* **= 17**	*p*-**Value**	*p*-**Value After Correction for Age**	**Age < 25** *n* **= 21**	**Age 25 – 30** *n* **= 9**	**Age > 30** *n* **= 27**	*p*-**Value**
Testosterone (nmol/L)										
Before 11:00 AM	14	6.2 [a]	5.7 (3.4-9.0)	3.3 (2.1-8.5)	0.4	0.1	6.5 (5.1-9.9)	3.7 [a]	3.3 (0.9-7.3)	0.3
After 11:00 AM	16	5.5, 4.0, 1.1 [a]	1.7 (0.7-4.3)	1.5 (0.9-6.5)	0.9	1	5.0, 7.7 [a]	4.7 [a]	1.1 (0.8-3.5)	0.2
LH (IU/L)										
Before 01-02-2019	30	5.6 (1.8-9.0)	2.6 (1.3-8.4)	2.1 (1.0-8.0)	0.2	0.3	3.9 (1.3-5.8)	2.0 (1.7-4.7)	3.9 (0.7-9.9)	0.0003
After 01-02-2019	3	NA [b]	2.8, 11.0	1.9			1.9, 2.8 [a]	NA [b]	11.0 [a]	
FSH (IU/L)										
Before 01-02-2019	31	40.4 (13.2-55.8)	13.3 (8.6-45.4)	13.0 (3.5-27.2)	0.01	0.01	13.5 (5.0-46.9)	11.4 (7.2-24.3)	14.7 (5.5-42.7)	0.001
After 01-02-2019	3	NA [b]	7.2, 58.0 [a]	4.8 [a]			4.8, 7.2 [a]	NA [b]	58.0 [a]	
SHBG (nmol/L)	28	68.0, 58.5, 44.7 [a]	27.1 (20.0-44.7)	24.4 (17.3-49.8)	0.08	0.2	16.2 (12.6-24.8)	23.9, 19.7 [a]	42.5 (24.4-57.0)	0.0001

Abbreviations: body mass index (BMI), follicle stimulating hormone (FSH), luteinizing hormone (LH), sex hormone binding globulin (SHBG), number of observations (*n*). Data are presented as median (IQR). Laboratory values for patients at baseline or during the last measurement available before the start of testosterone replacement therapy for LH, FSH, and testosterone. For SHBG the measurement closest to baseline was used. Values that were below the measuring threshold were considered equal to the measuring threshold to calculate the median and IQR. For example, when FSH was below 0.5, this was considered 0.5. For LH and FSH, measurements before and after the change in measuring method on 01-02-2019 are calibrated differently, therefore these measurements are described separately and a variable indicating whether the values were measured before or after 01-02-2019 was added to the model to calculate the *p*-value. *p*-values are calculated with age and BMI as continuous variables. [a] Individual measurements are given as there were too few observations to calculate a median and IQR. [b] There were no observations in this group.
